# An Optimized BP Neural Network Model and Its Application in the Credit Evaluation of Venture Loans

**DOI:** 10.1155/2022/8791968

**Published:** 2022-05-02

**Authors:** Mingkeng Chen, Xiaoyun Ma

**Affiliations:** ^1^Business School of Zhejiang Wanli College, Ningbo 315101, China; ^2^Business School, Jiyang College of Zhejiang A&F University, Shaoxing 311800, China

## Abstract

With the rapid development of entrepreneurship loans in China, the construction of a credit evaluation system of risk loans has become an important financial safeguard measure. This paper mainly studies the following three aspects. Firstly, in view of the subjective factors in the approval process of venture loans, based on the credit evaluation system of commercial banks and the data characteristics of venture loans, a credit evaluation system based on venture loans is constructed. Secondly, the randomized uniform design method is used to improve the population initialization method to realize the uniform distribution of the individual population. Finally, aiming at the problem of low efficiency of venture loan audit, this paper proposes an optimized BP neural network to evaluate the venture loan. Especially, through data processing, a credit index system is constructed, and then the optimized BP neural network model is determined in parameters. The model contains 15 input nodes, 1 hidden layer, and 2 output layers. Finally, the simulation shows that the optimized BP neural network model has obvious advantages in the loan evaluation. This paper includes the development status of credit evaluation of venture loans is empirically studied by using an optimized BP neural network model of nonexpected output.

## 1. Introduction

**Figure 1:**
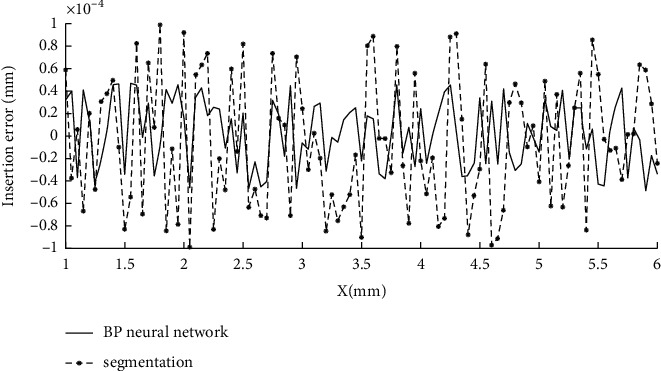
Error fluctuation comparison.

At present, the application approval process for venture loan is still that after the applicant submits the application, the relevant information of the applicant is manually reviewed by the approval staff, and finally, the risk assessment of the entrepreneur is made according to the subjective opinions of the approval staff, and the final loan amount is determined. This kind of approval method is not objective, and the approval result is easily influenced by subjective factors. In addition, when a large number of applicants submit their business applications at the same time, the approver needs to spend a lot of manpower to review them one by one, which leads to the situation that the waiting time is too long and the approval efficiency is low. The applicant may also be in the waiting process because the capital turnover is not timely, which leads to the difficulty of capital turnover and the failure of starting a business [1]. The venture loan policy has played an important role in supporting the employment of vulnerable groups and realizing the social support function. The effects brought by the implementation of the guarantee loan policy include economic effects, employment utility, and certain social effects. The pulling effects of the guarantee loan policy on employment are reflected in the multiplication effect of expanding the employment scale. At the same time, the support of entrepreneurship loans for entrepreneurship also has a significant impact on the change in employment structure. The employment ratio of the tertiary industry has greatly increased; the employment ratio of manufacturing and construction industries has decreased; and the employment structure of workers has been optimized. Entrepreneurship provides an important channel for interindustry transfer of labor force and an important force for industrial structure change and optimization [2]. The number of online lending platforms has increased sharply, and many platforms have overlapping business regions, market segments, and target customers, facing the same group of borrowers. In order to attract customers, some platforms adopt a “low threshold” response, which leads to repeated borrowing by borrowers. In order to make up for the shortcomings of their own credit capabilities, some platforms even attack the customers of leading platforms. Once the leading platform lends money to a certain customer, it will immediately pass the user's loan application to further amplify the repeated lending rate. The problem is likely to escalate, creating risks for the entire industry [3]. It can be seen from Figure 1 that the error fluctuation of the BP neural network algorithm is relatively small and the stability is higher. It adopts the error backpropagation learning algorithm to adjust parameters such as weights to make it optimal and has the advantages of wide adaptability and effectiveness [4]. Because the values of input nodes are uneven, in order to ensure that the neural network can learn samples better and prevent large number of information from drowning decimal information, all feature elements are normalized to −1∼1 and then input. Because the output of the activated S-type function cannot reach 0 or 1, set the label of the fuzzy block to 0.1 and the label of the clear block to 0.9 [5–9].

Some famous scholars began to study credit risk early, which can be traced back to the last century. Before the last century, the classical credit risk measurement method was mainly adopted, which mainly included the qualitative analysis of the bank's assets and liabilities, such as the expert rating system and credit rating system [10]. Let us take the situation of unsecured loans as an example. If the bank can collect the loan in full as scheduled, the bank can obtain regular interest and income. For example, if the risk of wind changes into actual loss, the probability ratio of occurrence is smaller, and the economic loss brought to the bank at this time is far higher than the interest and income. The asymmetric characteristics of the income distribution of credit risk make it impossible for us to assume that its probability distribution obeys the normal distribution when studying, which also brings some difficulties to the statistical analysis and measurement of credit risk. Therefore, the measurement management of credit risk has become a major challenge for the management of credit risk [11]. In the process of the financial exchange, the credit situation of the borrower is not as easy to observe as the market risk, that is, to know some credit situation of the borrower, it will not be as deep as the market risk, and after the financial exchange, the creditor has no way to supervise the borrower's capital use, management, and repayment intention. Usually, the amount of transaction information held by the power investigator and the borrower is different. In the financial and financial transaction, the creditor is facing the risk of economic loss and can only get a small amount of credit information from the borrower, which is a disadvantage [12]. Another important feature of nonsystematic credit risk is that the repayment ability of the lender will be influenced by some systematic factors such as macroeconomic background and inflation of goods, but under many circumstances, credit risk also depends on the influence of nonsystematic factors, such as the borrower's willingness to pay back the money, favorable risk, financial situation, the level of business management, and so on. Therefore, an important reason for credit risk management is to divide the risk through diversified investment [13, 14]. The novelty and contribution of the paper is that an optimized BP neural network model is used for risk assessment and forecasting for loans. This study combines the machine learning method with risk loan evaluation data and proposes and designs a risk loan credit evaluation model based on the optimized BP neural network. We need to build a system suitable for monitoring the credit of entrepreneurial loans. It can effectively improve the utilization rate of funds, and at the same time, it can improve the existing financial supervision system. The main contributions of this paper include the development status of credit evaluation of venture loans is empirically studied by using an optimized BP neural network model of nonexpected output. Furthermore, the correlation between the projection of production frontier and the dynamic change of credit evaluation development performance is analyzed.

## 2. Credit Evaluation of Venture Loan

At present, entrepreneurial loan services are provided all over the country. The low interest rate and low requirements of venture loans enable the masses to enjoy the benefits of the venture policy and meet the turnover needs of the majority of the masses for venture capital. Firstly, this chapter refers to the credit evaluation indexes of the three major commercial banks and preliminarily selects the credit evaluation indexes according to the actual situation of entrepreneurial loan data samples. Then, the preliminarily selected credit evaluation indicators are screened by the method of consistency ratio feature selection to remove redundant indicators. Finally, the data of the screened evaluation indexes are analyzed, and the weight distribution and assignment of the credit evaluation indexes of venture loans are made with reference to the evaluation index assignment methods of commercial banks and AHP, so as to complete the construction of the credit evaluation system of venture loans [15]. Today, with the rapid development of computer technology, the application of the Internet in China is more and more extensive, and online loans are also generated. However, the development speed of the credit evaluation system has not fully kept up with the development speed of Internet finance. Many online lending products mainly rely on the existing information of the platform itself or obtain a large number of users' data through other channels for evaluation. However, because the data obtained by each platform are not exactly the same, the credit evaluation system of each product has certain limitations [16]. The development of the venture loan is only about ten years ago. At present, personal credit evaluation still depends on subjective experience, and its information integrity is still insufficient compared with some commercial banks. Therefore, based on the personal credit evaluation model of commercial banks, combined with the credit evaluation methods and the data characteristics of venture loans, this paper establishes the credit evaluation index system of venture loans, which includes 20 indexes including personal category, economic category, family category, guarantee category, and credit category [17].

After the establishment of the preliminary credit evaluation index system, in order to make the credit evaluation index system more scientific and standardized, this paper uses the method based on consistency to screen the indicators and remove redundant indicators. As a representative feature selection method, CON is widely used in the selection of credit evaluation system indicators. The basic idea is: suppose a feature set *S*, if it meets the situation that some samples have the same values for the feature set *S* and the final results of these samples are close to the same, then it can be considered a good feature set [18]. Then, increase the number of indicators one after another, calculate the consistency ratio after the increase of indicators, and conduct modeling 20 times in total. The function of increasing indicators one after another is to compare the evaluation effect after the combination of multiple indicators. Observe the change of the consistency ratio of 20 modeling times, so as to screen redundant indicators and determine the final credit evaluation indicators. This method, considering the consistency ratio of each index, can also comprehensively consider the evaluation effect of multiple indexes and is a scientific index analysis method [19]. In this paper, the index assignment refers to the weight assignment method of the hierarchical analysis of the user credit evaluation system of rural credit cooperatives. Analytic hierarchy process (AHP), as a mainstream multiobjective analysis method of qualitative and quantitative analysis, has been widely used in many fields. The credit evaluation system in most countries adopts the AHP to analyze the index weight [20].

## 3. An Optimized BP Neural Network Model

### 3.1. Emergence of Particle Swarm Optimization Algorithm

PSO is an evolutionary algorithm based on swarm intelligence, ability, and behavior. Its main source comes from the behavior of flocks of birds, and the process of searching for food is abstracted. In the process of using this algorithm, some grains will be produced [21, 22]. The algorithm takes a bird colony as the modeling object, and each individual bird in the “population” is regarded as a “particle,” which represents a solution to the problem optimized by the algorithm, and all “particles” constitute the solution space of the problem.

This calculation method is very similar to the legacy calculation method in the overall search level. In this process, it is achieved through competition and cooperation among individuals. However, unlike the legacy algorithm, the parameter ratio used in the algorithm is simpler; the adjustment process is easier; and the overall ratio is simpler. After this algorithm was put forward, it soon occupied an important position in the computer domain; whether it is the optimization of function numbers or the system control system, it is better.

Particle swarm optimization algorithm in the domain of artificial intelligence and ability has great advantages [23].

(1) $$\begin{array}{c} f_{E}=\frac{1}{E}\\ =\frac{2}{{\left\|o_{k}\left(x_{p}\right)-y_{p}\right\|}^{2}}\\ =\frac{2}{\sum_{p}^{L}\sum_{k}^{m}{\left(o_{k}\left(x_{p}\right)-y_{p}\right)}^{2}}, \end{array}$$

where *O*_*k*_(*x*_*p*_) is the actual output value of the credit evaluation model and *y*_*p*_ is the expected output value of the credit evaluation model. The variables of *m* and *k* are the compute node number of the neural network algorithm.

In the searching process of the PSO algorithm, the particle makes the best solution for the individual and the best solution for the whole body in the newer process, but then the particle itself is updated, and there are two extreme points for different particles, and then the particle itself is updated. At each iteration, the “particle” will generate a new speed and direction and calculate the new individual fitness and global fitness. When the fitness meets the preset conditions, the iteration ends, and the global optimal solution to the problem is obtained at the same time.

When we use the particle swarm algorithm, we can analyze each particle and then perform a full-scale search during the search process. In this way, we will have a good value for each particle, which can be a good judgment of their direction and distance. In many spaces, to determine which solution is optimal, you can first find the best solution for each particle and then find the optimal solution for the entire population, so as to update the particles until the global optimal solution is found. The optimal solution is complete. The advantage of particle swarm optimization is to make the usage ratio simpler and the parameter ratio in the process smaller, but it is precise because of the smaller parameter ratio that we should be more careful when setting the parameters. In addition, there is no unusual and accurate theoretical support for this algorithm, and we usually judge it according to our own experience. The following is the setting of what kind of parameters are used to set up some proven parameters in the setting process [24]:

1. The number of seeds in the species group: Usually, the number of particles that we take is larger than or smaller than, but under normal circumstances, the choice can be completely used to solve many questions because, if there are more choices for fruit particles, the algorithm will take longer.
2. Grain size: Most of the time, when we choose the range of grain movement, we can set it according to different questions.
3. Maximum grain velocity: When the grain is flying, how far it can reach the final destination has a great relationship with its own speed. We need to set the speed, which can control the movement of the grain.
4. Acceleration coefficient: The parameters used in the learning process can be used with the values of using and waiting for most of them as follows.
5. The fitness function: For the particle swarm algorithm, the fitness function is usually less than the ratio.

### 3.2. Improved BP Neural Network Model Based on PSO Algorithm

Through the above analysis of the optimization capability of the particle swarm optimization algorithm, it can be found that the initial weight *ω* of the optimization algorithm has a great influence on the entire optimization process, and the value range of *ω* is [0, 1]. When *ω* is too large, the flying speed of the massless particles will increase, and the global search step size of the particles will also increase accordingly, thereby improving the convergence speed but will miss the global optimal solution and obtain the general solution. When it is too small, the flying speed of the massless particles is reduced, resulting in the reduction of *ω* of the global search step size, which improves the local search ability of refinement, but causes the particles to easily fall into the local optimal solution. It is difficult for the group to obtain the global optimal solution. In order to solve the influence of the weight *ω* on the PSO optimization algorithm, an improved BP neural network model is proposed based on the PSO algorithm. The improved BP algorithm enhances the local and global optimization capabilities of particle swarms by implementing weighted dynamics. The search process is constantly changing, and the error of the prediction result of the BP neural network prediction model is used as the fitness value to optimize the weights and thresholds of the BP neural network and improve the ability to predict the BP neural network model. The basic idea of PSO improving the BP algorithm is to analyze the fitness of each individual in the group to the environment, find the best location in the area where the individual is located, and use the cooperation and information sharing of each individual in the group to move to the best location in the area. The algorithm treats each individual in the swarm as a particle without volume and mass, and each particle flies in a given search space at a certain speed, thus realizing a dynamic iterative update [25, 26].

The optimized BP algorithm optimizes the initial weights and thresholds of the network. The optimized weights and thresholds are passed to the BP neural network for training and prediction, which avoids the network from falling to a local minimum during the training process, improves the training speed and learning ability of the network, and optimizes the performance of the network.

**Figure 2:**
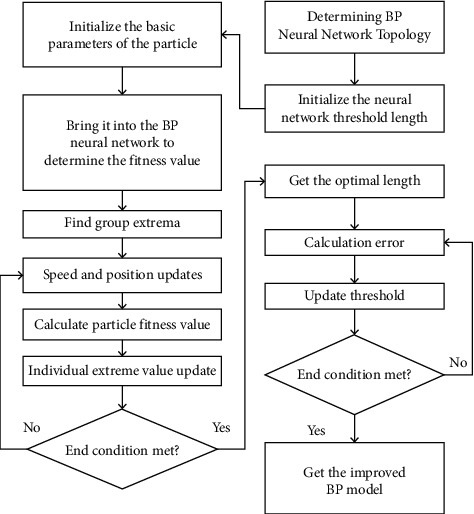
Optimized BP neural network flowchart.

The optimized BP neural network algorithm flowchart is shown in Figure 2, and its implementation process is discussed as follows:

Step 1: Determine the network structure of the optimized BP neural network.
Step 2: Initialize the weights and thresholds of BP and encode them to obtain the initial population.
Step 3: Initialize the parameters of the particle swarm algorithm: population size, particle dimension, learning factor, inertia weight, maximum number of iterations, velocity, and position.
Step 4: Select an appropriate fitness function to calculate the fitness value of each particle. The calculation formula is as follows, where *n* is the number of training samples and *N* is the population size.
  (2) $$\begin{array}{c} f\left(i\right)={\sum_{i=1}^{n}\left(y_{i}^{\prime }-y_{i}\right)}^{2}. \end{array}$$
Step 5: Compare the fitness value of each particle to find the individual optimal position and the global optimal position.
Step 6: Update the velocity and position of the particle according to the velocity and position update formula.
Step 6: Calculate the fitness value of the new particle and then update the individual optimal value and the global optimal value according to the fitness value.
Step 7: When the number of iterations is less than the maximum number of iterations, return to step 4; otherwise, end the iteration.
Step 8: Transfer the obtained optimal solution, that is, the optimal weights and thresholds optimized by the improved PSO algorithm, to the BP neural network for learning and training.

**Figure 3:**
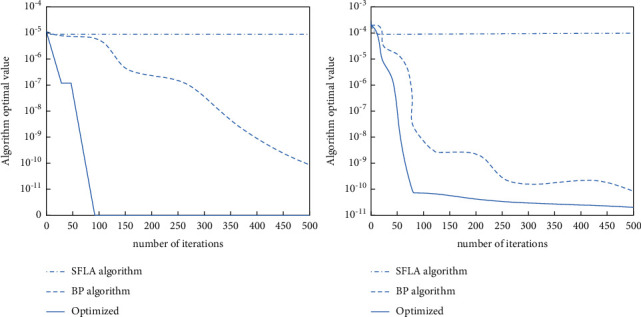
The iteration curve of several different kinds of models.

Figure 3 is the iterative curve of the optimized BP algorithm function. The optimization of the BP algorithm to realize the weight dynamics is mainly by using the scaling factor *λ* instead of the weight *ω*. The scaling factor utilizes the intrinsic relationship between the weight value and the learning factor and is represented by the nonlinear way of the learning factor. The scaling factor performs all functions on the update formula of the flight speed of the particle swarm to improve the optimization ability of the particle swarm algorithm convergence speed. The calculation formula of the scaling factor is shown in the following equation [27]:

(3) $$\begin{array}{c} \lambda =\frac{2}{\left|2-C-\sqrt{C^{2}-4C}\right|}, \end{array}$$

where *λ* is the scaling factor and *C* is a constant, and *C* > 4.

**Figure 4:**
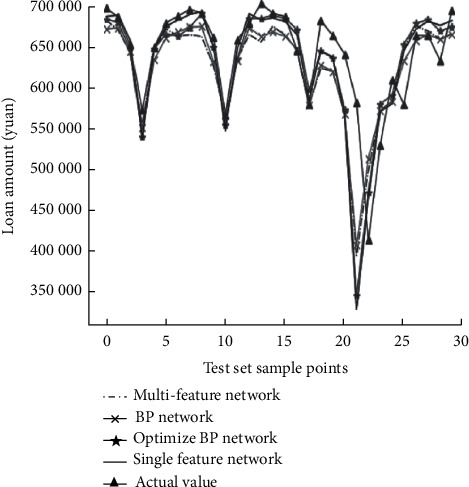
Results of several different kinds of models.

Figure 4 shows the prediction effect of the original BP neural network model and the optimized BP neural network model. In order to analyze the search ability of the optimized BP neural network model, we usually use the Sphere function number and Rastrigin function number during verification [28–35]. The Sphere function is the function used before. This function can be well verified for the local situation because this function has a large number of local extremums and is a continuous unimodal function. The search capability can be judged from a global perspective to find the best global solution.

## 4. Establishment and Solution of the Optimized BP Neural Network Model

In this paper, we try to use the proposed method to study credit risk. As we know, there are a lot of machine learning-based methods that are widely used in a lot of fields, such as genetic algorithm, K-means clustering algorithm, and so on [36–40]. However, in this paper, we only used the PSO-BPNN model to implement this work and do not consider other machine learning-based methods. There are many factors affecting credit risk, so in the process of data processing, it is necessary to carry out a weighted analysis of different influencing factors:

(4) $$\begin{array}{c} A={\left(a_{ij}\right)}_{n\times n}, \end{array}$$

where *a*_*ij*_ is equal to 1/*a*_*ij*_, that is, *a*_*ij*_ = 1.

Because each index has a different influence on enterprise credit risk, it is necessary to calculate their weights. We choose the correlation analysis method to calculate the three selected indicators. Firstly, the function correlation coefficient is defined as follows:

(5) $$\begin{array}{c} \rho =\frac{x_{i}U-\bar{x_{i}}U}{\sqrt{1/n\sum_{k=1}^{n}{\left(x_{k}-x_{i}\right)}^{2}\times \sqrt{1/n{\left(\sum_{k=1}^{n}\left(U_{i}-\bar{U}\right)\right)}^{2}}}}. \end{array}$$

This is the correlation coefficient of the custom function. The *ρ* is the function correlation coefficient.

Through calculation, the correlation coefficients among the three indicators are: input 0.44, output 0.39, and credit evaluation 0.33. Calculated by MATLAB, the standardized feature vectors of each index weight are as follows: input *w*_1_ = 0.43, output *w*_2_ = 0.28, and credit evaluation *w*_3_ = 0.17. Therefore, we establish the correlation model:

(6) $$\begin{array}{c} U=a_{1}f_{1}+a_{2}f_{2}+a_{3}f_{3}. \end{array}$$

*U* is the formula of the sum function, that is, three components are added together.

These three influencing factors are not comprehensive and reasonable enough. According to the results of the optimized BP neural network, it is not comprehensive enough to consider only the three factors of input, output, and credit evaluation. Therefore, when helping banks formulate credit strategies, we should also consider the influence of other factors, choose the TOPSIS method to prioritize the predicted value of enterprise credit risk, and process the data to get the information on the six influencing factors of known enterprises, such as profit, sales quantity, credit evaluation, number of input checks, number of output checks, and annual credit.

(7) $$\begin{array}{c} MSE=\frac{1}{n}\sum_{i=1}^{n}{\left({\hat{Y}}_{t}-Y_{i}\right)}^{2}, \end{array}$$

where *y*_*t*_ represents the independent variable of the *t*-th regression mode.

(8) $$\begin{array}{c} RMSD=\sqrt{\frac{1}{n}\sum_{t=1}^{n}{\left(\hat{y_{t}}-y_{t}\right)}^{2}}. \end{array}$$

This is an improved calculation result of the prediction result.

**Figure 5:**
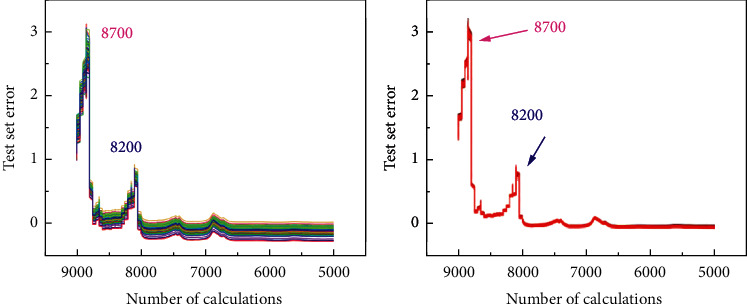
Error drop in model training.

Therefore, banks can first use the bank credit risk control model to calculate the threshold value. Once the threshold value is exceeded, they will not lend, and within the threshold value, they can lend. The data does not give the credit rating of each enterprise, so it is necessary to predict the credit rating of each enterprise first. It can be seen from Figure 5 that need to remove the amount of invalid data and then quantify the reputation rating. Then, the input amount and output amount after removing invalid data are selected as evaluation indexes.

## 5. Grasping and Processing of Data Sets

**Figure 6:**
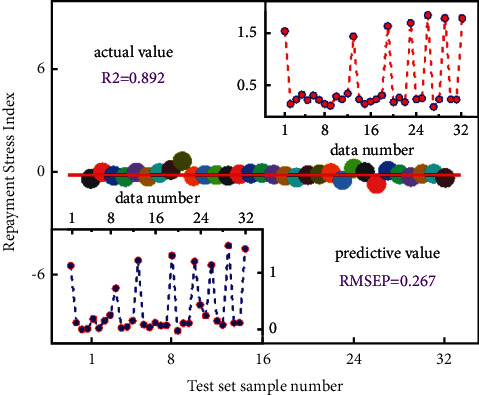
Difference between the predicted value and the true value.

AEXA is a network station with the largest number of URLs and the most detailed information release. However, it can be seen from Figure 6 that although its ranking data does not have absolute authority, many of its indicators have relative reliability, so we can refer to the ratio of indicators to data.

Because there is no absolute power in the online loan platform in front of China, the ranking results can be obtained either according to a certain index or according to the price evaluation scheme independently determined by the institutions themselves, and the ranking results of major institutions are not the same and cannot be fully included. This paper, according to the latest rating results distributed by the most powerful Institute of Finance and Finance, Chinese Academy of Chemical Sciences, and combined with the rating results of other more official parties, got the rating results of 23 platforms as the standard data.

(9) $$\begin{array}{c} f\left(x\right)=\sum_{i=1}^{n}\left(x_{i}^{2}-10\;\cos\left(2\pi x_{i}\right)+10\right). \end{array}$$

Use the semisupervised learning model to conduct the pretest. In the middle and early learning device, the optimized BP neural network model is adopted. Because in the optimized BP neural network model, models have different hidden layer node number *N* and different near neighbor number *K*, and one has four parameters, which cannot be trained at the same time. In the first group of parameter training, the number of nodes in the hidden layer is determined to train the near neighbor number. According to the training results of BP God's regression parameters through the network, it is determined that the *N* values of the number of nodes in the hidden layer of the two regression models are 2 and 4,respectively, and the *K* values of the number of neighbors of the two regression models are 2 to 10, so as to find the best combination of parameters to minimize MSE and RMSD.

(10) $$\begin{array}{c} N_{h}=\sqrt{N_{\text{in}}\times N_{\text{out}}}, \end{array}$$

where *N*_*h*_, *N*_in_, and *N*_out_ are the calculated values, input values, and output values, respectively.

It is more complicated to determine the divinatory symbols in the hidden layer because it can be judged by making use of the special points of learning, so we can analyze and judge according to some formulas.

(11) $$\begin{array}{c} N_{h}=\frac{N_{\text{in}}\times N_{\text{out}}}{2}. \end{array}$$

For the hidden layer, there is a need for a certain number of divinatory symbols, which are *N*_*h*_, *N*_in_, and *N*_out_. Show the number of input nerve cells and the number of output nerve cells.

## 6. Examples Based on the Optimized BP Neural Network

Firstly, the data of entrepreneurs are divided into training group and test group, and the training group data is modeled by BP neural network. After the model is obtained, the test group data is put into the model, and the predicted default rate of the test group data is obtained. According to the default rate, the default of each loan is predicted. Finally, the prediction accuracy of this algorithm is obtained according to the set evaluation criteria. The software used in the experiment is MATLAB.

This data set is the real data provided by credit customers when applying for bank loans or credit cards. At present, it is difficult to collect the basic data and materials for domestic credit evaluation. A total of 500 valid records were sorted out, and each record included 21 customer information fields. The customer information involved includes personal basic information (such as age, job type, housing, household registration, and current working hours), basic loan information (such as loan term, loan purpose, and loan amount), personal historical account data information (such as time deposit account data and current deposit account data), and so on. The 21st field is the bank's definition of customer credit rating, which is divided into two categories: “1” (normal repayment) and “0” (loan default).

**Figure 7:**
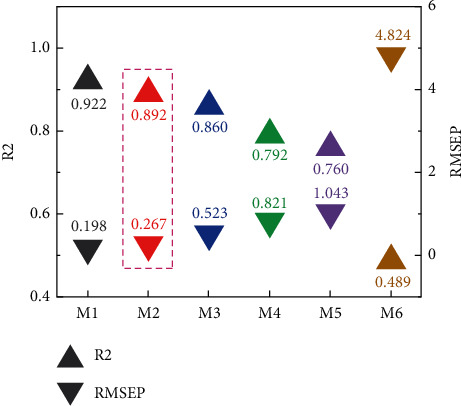
*R* ^2^ and RMSEP values for different models.

From the perspective of the business significance of commercial banks, the correct classification rate of default samples is the rate of successful early warning of actual default customers, and the improvement of this index can avoid losses for banks. The false positive rate is the rate of false warning to normal or high-quality customers, which affects the mutual trust relationship between banks and customers. Therefore, these two indicators are particularly important. The proposed method has good performance in these two indexes. It can be seen from Figure 7 that in order to facilitate business processing, most of the bank's data are stored in different electronic forms in blocks. It seems that it is not difficult to extract data that are loyal to reality, standardized in format, and convenient for application from the massive data of various forms, but in fact, the workload is huge. In the process of introducing advanced intelligent algorithms for data mining, data sorting and preprocessing will also be the difficulties.

Machine learning has prominent advantages such as reliability, efficiency, accuracy, and safety and is of great value to the development of human society. Compared to machines, brain size has a limited effect on thinking. Humans can only concentrate on 3–4 things at most at once, and machines have thousands of times more processing power. Machine learning plays an integral role in many stages of the financial ecosystem, from loan approval to asset management to risk assessment. Based on the results of this study and the advantages of machine learning, we believe that machine learning has important application value in the credit evaluation of risky loans. The neural network method used in this study has a certain reference value.

## 7. Conclusion

In this paper, the machine learning method is applied to the risk loan evaluation data; thus, a risk loan credit evaluation model based on the neural network is proposed and designed. The evaluation method of the credit risk of enterprises and the credit risk control model can be widely applied to the investigation of making credit strategies by banks and other financial fields. An optimized BP neural network model is used to predict default risk. The network structure is simple; the convergence speed is fast and easy to realize; the prediction accuracy is high; and the capacity of parameters and samples is reduced. Using particle swarm optimization to optimize the vector machine model is suitable, practical, simple, and easy to solve. The model used in this paper is to select the appropriate model and statistical method as far as possible according to the characteristics and problem requirements of the data. According to the algorithm corresponding to each model and its own characteristics, machine learning methods are widely used for data processing to make the results more scientific and visual. It has the strong adaptive ability and strong fault tolerance and can better deal with multiclassification problems. Compared with other methods, the method proposed in this study has the strong suitability, accuracy, and efficiency in the analysis of China's economic situation. However, the application effect of larger data needs further verification. The results of this study have important practical significance for promoting the application of machine learning in bank loan risk assessment. The model based on neural networks is expected to be popularized in the banking system.

## Data Availability

The data used to support the findings of this study are available from the corresponding author upon request.
